# Supplementary material for: Assessing the reporting of Dengue, Chikungunya and Zika to the National Surveillance System in Colombia from 2014–2017: A Capture-recapture analysis accounting for misclassification of arboviral diagnostics
Source: PLoS Negl Trop Dis. 2021 Feb 4;15(2):e0009014. doi: 10.1371/journal.pntd.0009014 (PMC7888590; doi:10.1371/journal.pntd.0009014)
Supplement: S1 Appendix — (PDF) [file pntd.0009014.s001.pdf]

Assessing the reporting of Dengue, Chikungunya and Zika to the National Surveillance System in Colombia from 2014-2017: A Capture-recapture analysis accounting for misclassification of arboviral diagnostics.

Carabali M, Jaramillo GI, Rivera VA, Mina NJ, Restrepo BN, Zinszer K.

Appendix 1. International Disease Codes and Surveillance codes used in the study.

Table S.1A. International Disease Codes for Diagnosis -10 version (IDC-10), Included on Institutional Data Registries.

CIE-10 Code	Description
**A689**	Relapsing fever, unspecified
**A778**	Other spotted fevers
**A779**	Spotted fevers, unspecified
**A90/ A90X**	**Dengue fever [classical dengue]**
**A91/A91X**	Dengue hemorrhagic fever
**A920**	Chikungunya virus disease; Chikungunya (hemorrhagic) fever
**A928**	Other specified mosquito-borne viral fevers
**A929**	Mosquito-borne viral fever, unspecified
**A93**	Other arthropod-borne viral fevers, not elsewhere classified
**A94X**	Unspecified arthropod-borne viral fever, Arboviral fever; Arbovirus infection
**A98**	Other viral hemorrhagic fevers, not elsewhere classified
**A985**	Hemorrhagic fever with renal syndrome
**A99**	Unspecified viral hemorrhagic fever
**R500**	Fever and chills, unknown
**R501**	Persistent fever
**R509**	Fever, unspecified
**A925**	Zika virus disease

Table S.1B. Surveillance Diagnosis Codes Included on SIVIGILA data Registries.

Code	Description
**210**	Dengue (with and without warning signs)
**220**	Severe Dengue
**217**	Chikungunya

**895**	Zika

Table S.2. Case report form (CRF)

I. General Information

**1. Administrative Data**			
**Variable Name**	***Description***	**Variable Name**	**Description**
ID Number	*Official identification number*	ID Type	*Type of document: (CC=citizenship, TI=Identity Card, RN=Birth certificate)*
Medical Registry Number	*Number used by the institution to identify the patient*	Type of Insurance	*According to the Colombian system, subsidized, contributory, special, etc*
EPS Code	*Insurer provider code*	EPS Name	*Insurer provider name*
IPS Code	*Institution NIT/Code*	IPS Name	*Institution Name*
Date of consultation	*DD/MM/YYYY*	Level of Attention	*Level of complexity, 1, 2, 3 or 4*
**2. Sociodemographic**			
**Variable Name**	**Description**	**Variable Name**	**Description**
Age	*Age of the patient*	Unit	*Months, years*
Sex	*Female/Male*	If women, pregnant?	*Yes or No*
Ethnicity	*Afro Colombian. Indigenous, White, other*	Code Province	*Official code of the province*
Neighborhood	*Name of the neighborhood*	Code City	*Official code of the city*
Address	*If available, complete residential address*	Area	*Rural or Urban*
Occupation	*Main activity registered*		
**3. Medical condition**			
**Variable Name**	**Description**	**Variable Name**	**Description**
Reason to Consult	*Patient's motive of consultation*	Diagnosis at entry	*Doctor's diagnostic at entry*
Onset of Symptoms	*Date of onset of symptoms DD/MM/YYYY*	Duration of Symptoms	*Number of days referred by the patient*
Clinical Diagnosis	*Doctor's Primary Diagnosis*	Clinical Diagnosis 2	*Doctor's Secondary Diagnosis*
Suspected Arboviruses	*Yes or Not, as indicated in clinical chart*	Type of Arbovirus	*Dengue, Chikungunya and Zika*
Require Treatment	*Yes or Not, as indicated in clinical chart*	Procedure/Treatment	*Description of treatment indicated*

IPD/OPD	*OPD=Ambulatory-Outpatient IPD=hospitalized-Inpatient*	Date of Hospitalization	*DD/MM/YYYY*
Final Status	*Live or death*	If dead, date of death	*DD/MM/YYYY*
Final Diagnosis	*Final primary Diagnosis (at discharge)*	Final Classification	*Confirmed or suspected final diagnosis*
**4. Laboratory confirmation**			
**Variable Name**	***Description***	**Variable Name**	**Description**
Laboratory Tests	*Any performed lab tests? Yes or No*	Date of laboratory testing	*DD/MM/YYYY*
RDT	*Yes or No*	RDT Result	*Positive; Negative; non-Conclusive*
IgM	*Yes or No*	IgM Result	*Positive; Negative; non-Conclusive*
IgG	*Yes or No*	IgG Result	*Positive; Negative; non-Conclusive*
RT-PCR	*Yes or No*	RT-PCR Result	*Positive; Negative; non-Conclusive*
**I. Clinical and additional laboratory information**			
**Variable Name**	***Description***	**Variable Name**	**Description**
Fever	*Yes or No*	Abdominal Pain	*Yes or No*
Myalgia	*Yes or No*	Rash	*Yes or No*
Arthralgia	*Yes or No*	Arthritis	*Yes or No*
Headache	*Yes or No*	Conjunctivitis	*Yes or No*
Hemorrhagic manifestations	*Yes or No*	Shock	*Yes or No*
**Variable Name**	***Description***	**Variable Name**	**Description**
Leukocytes (x 10^3/uL)	*Count x 103/uL*	Hemoglobin (g/dL)	*Units* (g/dL)
Platelets (x 103/uL)	*Count x 103/uL*	Hematocrit (%)	*Percentage (1-100)*
AST (u/L)	*Units (u/L)*	ALT (u/L)	*Units (u/L)*
