# Supplementary material for: Assessing the reporting of Dengue, Chikungunya and Zika to the National Surveillance System in Colombia from 2014–2017: A Capture-recapture analysis accounting for misclassification of arboviral diagnostics
Source: PLoS Negl Trop Dis. 2021 Feb 4;15(2):e0009014. doi: 10.1371/journal.pntd.0009014 (PMC7888590; doi:10.1371/journal.pntd.0009014)
Supplement: S2 Appendix — (PDF) [file pntd.0009014.s002.pdf]

# Assessing the reporting of Dengue, Chikungunya and Zika to the National Surveillance System in Colombia from 2014-2017: A Capture-recapture analysis accounting for misclassification of arboviral diagnostics.

## Appendix 2. Case Definitions, according to the National Surveillance Program (SIVIGILA)(1-3):

### Dengue

Probable case: Patient from an endemic area who meets the definition of a dengue case (with or without warning signs).

Dengue without warning signs: Acute febrile illness of 2 to 7 days of evolution in which two or more of the following manifestations are observed: headache, retro-ocular pain, myalgias, arthralgia, rash, rash or leukopenia.

Dengue with warning signs: Patient who meets the above definition and that during the effervescence period (remission of fever) presents also at least one of the following warning signs: Intense and continuous abdominal pain or pain on palpation, persistent vomiting, diarrhea, fluid accumulation (ascites, pleural effusion, pericardial effusion), mucosal bleeding, lethargy or irritability (mainly in children), postural hypotension, painful hepatomegaly> 2 cm, temperature drop, abrupt platelet drop (<100,000) associated with hemoconcentration.

Probable case of severe dengue: Any case of dengue that meets one or more of the following manifestations:

- Severe plasma extravasation: leading to dengue shock syndrome or fluid accumulation with respiratory distress.
- Severe hemorrhages: Patient with acute febrile illness, who presents severe hemorrhages with hemodynamic compromise.
- Severe organ damage: Patient with acute febrile disease and who presents clinical or paraclinical signs of severe organ damage such as: liver damage, central nervous system damage, heart or other involvement of another organ.

Confirmed case of dengue: A probable case of dengue, severe dengue, or dengue mortality confirmed by any of the laboratory criteria for the diagnosis of dengue. PCR or viral isolation in patients with less than 5 days of onset of fever or IgM Dengue ELISA test in patients with 5 or more days of onset of fever (rapid tests are not accepted, its use to date is aimed at clinical management of the patient, but not for confirmation or dismissal of cases). Confirmation is also granted in presence of antigen detection by NS1 ELISA and/or in presence of seroconversion of IgM or IgG ELISA (including four-fold increased DENV titers) in paired samples.

**Chikungunya**

Suspected case of chikungunya**:** Patient who resides or has visited from 8 to 15 days before the onset of symptoms, a municipality located between 0 and 2,200 meters above sea level, where chikungunya **have not been** confirmed by laboratory, and which present fever greater than 38°C, arthralgia severe or acute onset arthritis, erythema multiform or symptoms that are not explained by other medical conditions.

Clinically confirmed case of chikungunya: Patient who resides or has visited from 8 to 15 days before the onset of symptoms, **an endemic municipality** located between 0 and 2,200 meters above sea level, where chikungunya viral circulation have been confirmed by laboratory and which present fever greater than 38°C, arthralgia severe or acute onset arthritis, erythema multiform or symptoms that are not explained by other medical conditions.

Laboratory confirmed case of chikungunya: A probable case with any of the following laboratory tests specific for the virus with a positive result (viral isolation, RT-PCR, IgM), or four-fold increase in the IgG antibody titers specific for chikungunya virus in paired samples by a difference of 15 days between the taking of these.

**Zika**

Suspected case of Zika: Patient who resides or has visited from 8 to 15 days before the onset of symptoms, a municipality/country located between 0 and 2,200 meters above sea level without

confirmed zika circulation who presents non-purulent conjunctivitis or conjunctival hyperemia, pruritus, arthralgia, myalgia, headache or malaise.

Clinically confirmed case of Zika: Patient who belongs to a population at risk (>65 years of ages, pregnant women, neonates, children under one year of age and people with comorbidities) and /or general population from municipalities **with laboratory confirmed ZIKV transmission**, presenting rash and elevation of axillary body temperature greater than 37.2 degrees centigrade, and one or more of the following symptoms that are not explained by other medical conditions: non-purulent conjunctivitis or conjunctival hyperemia, pruritus, arthralgia, myalgia, headache or malaise, and that has been in places less than 2200 meters above sea level.

Laboratory Confirmed case of Zika: Suspected case with a positive result for ZIKV using ZIKV RT-PCR, performed at the National Virology Reference Laboratory of the National Laboratory Network of the National Institute of Health, or collaborating centers designated by the INS. Confirmed cases should have followed the diagnosis algorithm where first, DENV and CHIKV are ruled-out.
