# Supplementary material for: Assessing the reporting of Dengue, Chikungunya and Zika to the National Surveillance System in Colombia from 2014–2017: A Capture-recapture analysis accounting for misclassification of arboviral diagnostics
Source: PLoS Negl Trop Dis. 2021 Feb 4;15(2):e0009014. doi: 10.1371/journal.pntd.0009014 (PMC7888590; doi:10.1371/journal.pntd.0009014)
Supplement: S3 Appendix — (PDF) [file pntd.0009014.s003.pdf]

Assessing the reporting of Dengue, Chikungunya and Zika to the National Surveillance System in Colombia from 2014-2017: A Capture-recapture analysis accounting for misclassification of arboviral diagnostics.

Appendix 3A. Capture-recapture analysis following Vong's et al., approach.

Table S.3A. Estimation of overall -unadjusted- total number of cases, and multiplication factors for notification by city.

City	Capture (N_A)	Recapture (N_B)	Reported (X_{AB})	Estimated Total cases (N)	Multiplication Factor (MF)
**Cali**	97,193	75,379	11,223	652,808.7	8.7
**Medellin**	32,437	28,689	4,760	195,512.9	6.8
**Villavicencio**	18,121	14,734	961	277,863.4	18.9

N is the estimated total number of arboviruses cases $N = \left[\frac{(N_A+1)(N_B+1)}{X_{AB}}\right] - 1$
N_A is the number of cases by the capture (from collaborating institutions), N_B is the number of cases by the recapture (cases notified in SIVIGILA), and X_{AB}, is the number of cases reported by both, the capture and recapture. The multiplication factor (MF) is the inverse of underreporting rate and it is estimated by: $MF = N/N_B$

Table S.3B. Unadjusted multiplication factors for notification of arboviral conditions by site.

**Diagnostic**	**Multiplication Factors (MF)**		
**Site**	**Cali**	**Medellin**	**Villavicencio**
Dengue	2.5	5.9	2.0
Chikungunya	38.2	8.6	4.3
Zika /other vector-borne diseases	2.7	0.7	110.6

Appendix 3B. Reporting by health care and predicted probabilities by city using the adjusted model.

Table S.4. Factors associated to reporting/Notification of arboviral diseases in Cali (Colombia) 2014-2017: Stratified Analysis by health care provider.

**Type of provider in Cali, Colombia**	**Subsidized Institution**		**Contributory Institution**	
	N= 13,447		N=83,740	
**Variable**	**RR**	**[95% CI]**	**RR**	**[95% CI]**
<18 Years old	Ref.	-	Ref.	-
> 18 Years old	1.24	[1.13, 1.37]	5.62	[5.19, 6.1]
**Year**				
2014	Ref.	-	Ref.	-
2015	1.28	[1.12, 1.45]	1.11	[1.02, 1.21]
2016	1.57	[1.38, 1.79]	2.68	[2.47, 2.91]
2017	0.3	[0.22, 0.42]	2.2	[2.01, 2.41]
**Institutional Diagnosis**				
Undifferentiated fever	Ref.	-	Ref.	-
DENV	11.61	[10.65, 12.66]	3.4	[3.22, 3.59]
CHIKV	1.04	[0.74, 1.46]	1.28	[1.18, 1.38]
Non-specified vector-borne diseases	5.94	[4.84, 7.29]	2.32	[2.17, 2.47]

Table S.5. Overall predicted probability of reporting by institutional diagnostic in each city.

**Site**	**Cali (Subsidized)**	**Cali (Contributory)**	**Medellin**	**Villavicencio**
**Diagnosis**	**%, (95% CIs)**	**%, (95% CIs)**	**%, (95% CIs)**	**%, (95% CIs)**
Undifferentiated fever	6.0 (5.6, 6.5)	6.1 (5.8, 6.4)	8.7 (8.4, 7.1)	2.4 (2.2, 2.7)
DENV	70.1 (66.7, 73.5)	20.7 (20.1, 21.7)	47.6 (46.1, 49.2)	21.9 (19.6, 24.3)
CHIKV	6.3 (4.2, 8.3)	7.8 (7.3, 8.2)	28.7 (18.5, 38.9)	10.2 (7.4, 12.9)
Non-specified vector-borne diseases	35.8 (28.9, 42.7)	14.1 (13.5, 14.6)	45.3 (36.6, 54.1)	6.3 (4.9, 7.6)

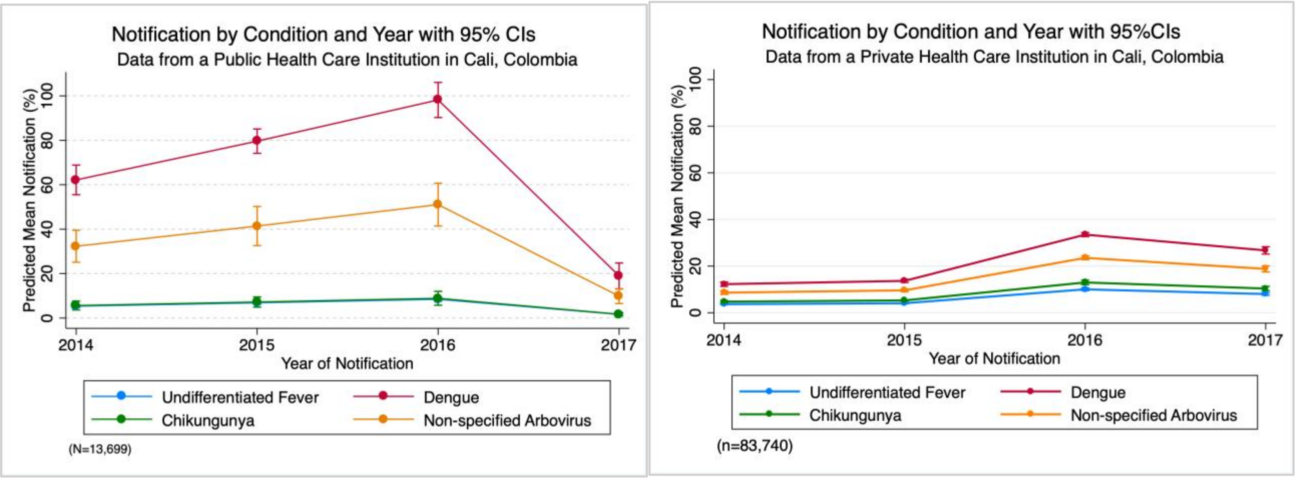

Figure S1. Overall predicted probability of reporting by institutional diagnostic, stratified by health care provider in Cali.
