# Supplementary material for: Assessing the reporting of Dengue, Chikungunya and Zika to the National Surveillance System in Colombia from 2014–2017: A Capture-recapture analysis accounting for misclassification of arboviral diagnostics
Source: PLoS Negl Trop Dis. 2021 Feb 4;15(2):e0009014. doi: 10.1371/journal.pntd.0009014 (PMC7888590; doi:10.1371/journal.pntd.0009014)
Supplement: S4 Appendix — (PDF) [file pntd.0009014.s004.pdf]

Assessing the reporting of Dengue, Chikungunya and Zika to the National Surveillance System in Colombia from 2014-2017: A Capture-recapture analysis accounting for misclassification of arboviral diagnostics.

Appendix 4. Simulation Extrapolation for Misclassification results (MC-SIMEX).

To estimate the effect of the misclassification on the estimation of reporting rates, we used the simulation extrapolation method for misclassification (MC-SIMEX)(4, 5). We used the R package 'simex', which provides functions to use the MC–SIMEX method on our regression-based analysis, to produce graphics and summary statistics of the corrected estimates. Simplifying, the MC-SIMEX method works by 1) a simulation step, adding a degree of measurement error to the naïve estimates (observed data), according to the provided information of specific/sensitivity of the observations; and 2) extrapolating back the estimates to the point of not measurement error (4-6). The MC-SIMEX procedure for this study included the use of three sets of correlation matrix for each city: 1) a low sensitivity/specificity matrix were we set the correlation matrix between main diagnosis to 70%; 2) a high sensitivity/specificity matrix were we set the correlation matrix between main diagnosis to 90%; and 3) a correlation matrix using the observed data, this is, the actual correlation observed between the notified diagnosis and the institutional diagnosis, having the clinical diagnosis as the reference (Table S.4).

Table S.6. Naïve and corrected estimates (log scale) from the Simulation Extrapolation for misclassification of the arboviral diagnosis in Colombia.

City/Diagnosis	Naïve Estimates	Corrected Estimates MC-SIMEX		
		70%	90%	As observed
**Cali**	**log(rate)**	**log(rate)**	**log(rate)**	**log(rate) [correlation]**
Dengue	1.28	1.98	1.84	2.36 [64%]
Chikungunya	0.2	0.13	0.12	0.53 [65%]
Non-Specified VBD	0.88	1.58	1.46	2.06 [63%]
**Medellin**				
Dengue	1.7	2.57	2.41	3.33 [49%]
Chikungunya	1.19	2.65	2.57	3.14 [14%]
Non-Specified VBD	1.65	3.95	3.87	4.47 [16%]

Villavicencio				
Dengue	2.2	3.47	3.28	4.19 [54%]
Chikungunya	1.43	2.64	2.52	3.25 [40%]
Non-Specified VBD	0.94	1.46	1.38	1.90 [47%]

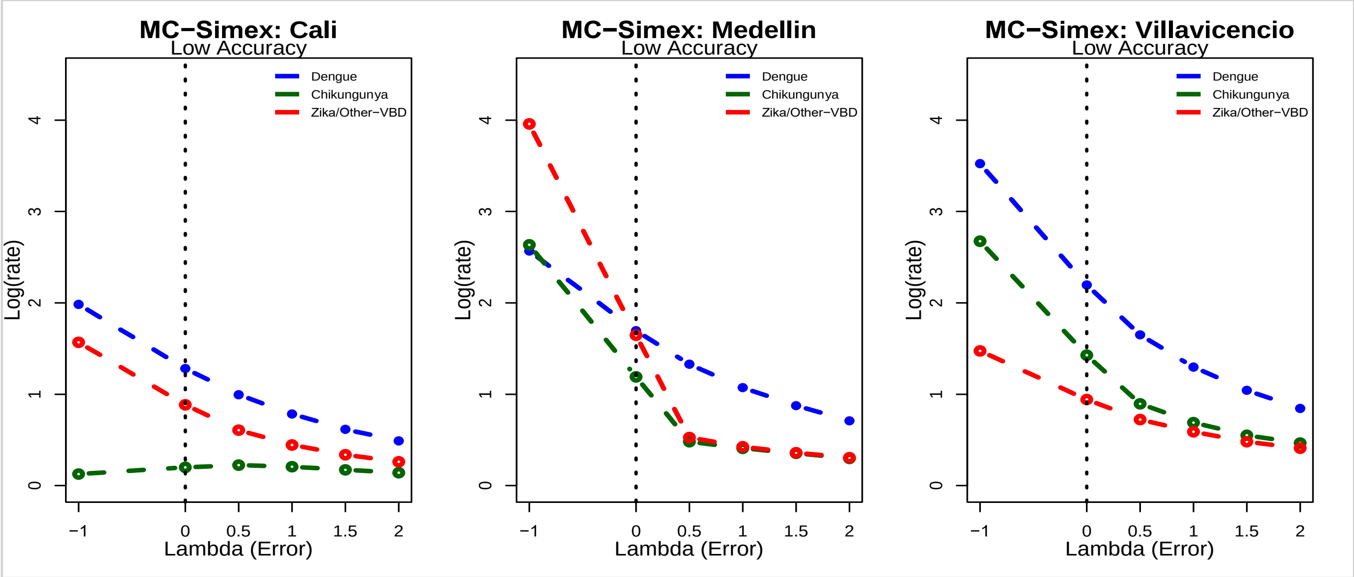

Figure S.2. Measurement Error estimation of the reporting/notification by condition, adjusted by age and year of notification in Cali, Medellin, and Villavicencio (Colombia) 2014-2017. Simulation Extrapolation results using a low accuracy (70%) of the clinical diagnosis.

References:

1. Instituto Nacional de Salud (INS). Protocolo de Vigilancia en Salud Pública, Dengue (Surveillance Protocol in Public Health, Dengue). . In: Instituto Nacional de Salud (INS) C, ed. *Surveillance*. Santafé de Bogota, Colombia. : Instituto Nacional de Salud (National Institute of Health). , 2014:19.
2. Instituto Nacional de Salud (INS). Protocolo de Vigilancia en Salud Publica Enfermedad por Virus Zika. In: Instituto Nacional de Salud (INS), ed. Bogota, Colombia: VIGILANCIA Y ANÁLISIS DEL RIESGO EN SALUD PÚBLICA, 2017:22.
3. Instituto Nacional de Salud (INS). Protocolo de Vigilancia en Salud Publica Enfermedad por Virus Chikungunya. In: Instituto Nacional de Salud (INS), ed. Bogota, Colombia: VIGILANCIA Y ANÁLISIS DEL RIESGO EN SALUD PÚBLICA, 2017.
4. Küchenhoff H, Mwalili SM, Lesaffre E. A General Method for Dealing with Misclassification in Regression: The Misclassification SIMEX. *Biometrics* 2006;62(1):85-96.
5. Lederer W, Küchenhoff H, Lederer MW, et al. Package 'simex'. 2019.
6. Lederer W, Küchenhoff H. A short Introduction to the SIMEX and MCSIMEX. *The Newsletter of the R Project Volume 6/4, October 2006* 2006;6:26.
