# Supplementary material for: Assessing the reporting of Dengue, Chikungunya and Zika to the National Surveillance System in Colombia from 2014–2017: A Capture-recapture analysis accounting for misclassification of arboviral diagnostics
Source: PLoS Negl Trop Dis. 2021 Feb 4;15(2):e0009014. doi: 10.1371/journal.pntd.0009014 (PMC7888590; doi:10.1371/journal.pntd.0009014)
Supplement: Attachment — Submitted filename: PNTD-D-20-00239_responsetoreviewers_201120.pdf [file pntd.0009014.s007.pdf]

November 20, 2020

Dr. Brianna R. Beechler,
Guest Editor
Dr. Sylvie Alonso,
Deputy Editor
PLOS Neglected Tropical Diseases

Dear Dr. Beechler, Dr. Alonso, and editorial committee,

Thank you for considering and reviewing our manuscript entitled "*Assessing the reporting of Dengue, Chikungunya and Zika to the National Surveillance System in Colombia from 2014-2017: A Capture-recapture analysis accounting for misclassification of arboviral diagnostics*." PNTD-D-20-00239) in your journal.

We reviewed our manuscript and answered the recommendation from the reviewers accordingly. Below we provide our response to the reviewers and attached the two revised versions of the document, as requested by the journal.

Sincerely,

Mabel Carabali, on behalf of the co-authors.
************************

Reviewer comments:

**Reviewer #1**: The expansion of the methods section has made the paper much clearer. Rather than calling the multiplication factor in lines 221-222 the inverse of the underreporting rate perhaps call it a reporting rate. Also, maybe say here what is a considered a good or bad value for this.

**Answer:** Thank you for your comment, we have revised the paragraph and rephrased as follows:

*"The multiplication factor (MF) is the inverse of underreporting rate and it is estimated by: ($MF=N/N_B$) and is the number by which the surveillance- reported cases needs to be multiplied in order to estimate a relatively valid-true total number of cases, assuming representativeness of the reported cases to the surveillance system [38, 39, 41, 47-50]. In the literature MFs ranges from 1 to 28 for dengue cases according to the year of notification and the type (sever vs non-severe) and could be higher for other emergent diseases [40, 47-52]."*
