# Supplementary material for: Assessing the reporting of Dengue, Chikungunya and Zika to the National Surveillance System in Colombia from 2014–2017: A Capture-recapture analysis accounting for misclassification of arboviral diagnostics
Source: PLoS Negl Trop Dis. 2021 Feb 4;15(2):e0009014. doi: 10.1371/journal.pntd.0009014 (PMC7888590; doi:10.1371/journal.pntd.0009014)
Supplement: Attachment — Submitted filename: PLOS Review.docx [file pntd.0009014.s005.docx]

**Overall impression:**

The paper explores an important and well-known problem with dengue, chikungunya and Zika in Latin America. I liked the choice of comparing 3 different cities with varying levels of endemicity and thought that the concept of exploring under-reporting by sex, age, year and healthcare provider was interesting. However, I felt this concept wasn’t fully utilized. If under-reporting in respect of sex, age, year and healthcare provider had been explored for each pathogen then targeted suggestions could have been created. I feel it is really important to separate these factors by pathogen due to their differing host demographics. The paper would benefit from some clear hypotheses to investigate. This would then structure the result and discussion sections better for easier reading. The main concern for the paper is that the methodology is not clearly explained and I am not sure of the choice of statistical testing. This paper is well worth revising for resubmission as it would be interesting to see the concepts of the paper fully explored.

| **Major Criticisms:** | |
| --- | --- |
| Line | Comment |
|  | The choice for statistical testing is unusual, potentially being an inappropriate choice. It is not fully explained, making evaluation difficult. The authors use a Poisson regression on a binary outcome predictor (recaptured or not)? More appropriate to consider a logistic regression with a binomial link function or a Poisson regression on the number of reported cases, with the number available as an offset.  I also think the presentation of the MC-SIMEX analyses is not ideal, it is presented as a magic black box. What does it do? How does it work? What are the assumptions? Please could you also clearly state the parameters used for the model. |
|  | Hypotheses are required to structure the paper. This will particularly help with the results and discussion sections. Specifically, it would be interesting to ask the question why the datasets are different. It is not clear from the paper whether the dominant hypothesis is that under-reporting is due to poor reporting to the national surveillance system, clinical diagnosis is poor or whether cross-reactivity/multiple infections are responsible.  In the methods section, I would like to see the expectations clarified. Do we expect the datasets to be identical if reporting has worked properly? Or is there something more complicated with the laboratory testing process? If a patient goes to a different healthcare facility, will it be reported in the 'recapture' but not the capture? |
|  | The wording ‘capture-recapture study’ invokes a specific type of analysis, so was surprised to see only a Poisson regression in the methods section. |

| **Minor Criticisms:** | |
| --- | --- |
| Line | Comment |
| Introduction | |
| 72 | Specifically, they are mainly carried by *Aedes aegypti and Aedes albopictus*. |
| 81 – 84 | As you can get severe joint pain with dengue; you can get headaches with all 3 etc. It would be good if you could include the sentence that many of these symptoms aren’t differential. |
| 88 – 89 | What is known about immunity to Zika post infection? |
| 89 – 91 | Mention dengue haemorrhagic fever. |
| 109 – 112 | Oddly phrased sentence, specificity should never change irrespective of prevalence. Also, it might be a good point to bring up cross-reactivity between dengue and Zika reducing the accuracy of testing. |
| Methods | |
| 128 – 129 | Maybe very brief description (a few words) what capture-recapture is here. |
| 133 – 145 | It would be useful to put the Zika and chikungunya incidence alongside dengue for each of the cities. If it doesn’t read easily, perhaps do it as a table? |
| 166 – 171 | I’m not certain whether the surveillance data that you are using for your recaptured data set is purely confirmed cases or whether they also include probable cases which have been notified to SIVIGILA. |
| Discussion | |
| 296 – 310 | Any theories why the 2^nd^ largest city has the most dengue cases? Are there any environmental differences between the cities? |
|  | It might be helpful to have some line graphs of dengue, chikungunya and Zika outbreaks so that we can see which years are dominated by which virus. |
| Appendix | |
| Appendix 3 | Perhaps define what a subsidized and contributory institution is. |
