# Supplementary material for: Assessing the reporting of Dengue, Chikungunya and Zika to the National Surveillance System in Colombia from 2014–2017: A Capture-recapture analysis accounting for misclassification of arboviral diagnostics
Source: PLoS Negl Trop Dis. 2021 Feb 4;15(2):e0009014. doi: 10.1371/journal.pntd.0009014 (PMC7888590; doi:10.1371/journal.pntd.0009014)
Supplement: Attachment — Submitted filename: PNTD-D-20-00239_responsetoreviewers_290920.docx [file pntd.0009014.s006.docx]

September 29, 2020

Dr. Brianna R. Beechler,

Guest Editor
Dr. Sylvie Alonso,
Deputy Editor
**PLOS Neglected Tropical Diseases**

Dear Dr. Beechler, Dr. Alonso, and editorial committee,

Thank you for considering and reviewing our manuscript entitled “*Assessing the reporting of Dengue, Chikungunya and Zika to the National Surveillance System in Colombia from 2014-2017: A Capture-recapture analysis accounting for misclassification of arboviral diagnostics*." PNTD-D-20-00239) in your journal.

We appreciate the opportunity to review our manuscript based on the recommendations and comments from the reviewers, which we consider that greatly improve the quality and readability of our manuscript. Below we provide a detailed list of our responses to the reviewers, indicating where and how the changes were made in the body of our manuscript or supplementary documentation, and attached the two revised versions of the document, as requested by the journal.

Sincerely,

Mabel Carabali, on behalf of the co-authors.

**Editorial notes:**

****Reviewers comments are in italics and grey****

*All three reviewers mentioned that better description of data sources and analysis techniques were needed in the methods section, to better understand the results. Additional concerns about the statistical analyses were raised by multiple reviewers - please consider these comments in your revised version and if you feel that changes to your analyses are not required please justify why. Additionally, please clarify whether data from the same hospital/region can be considered "independent" and if not then if a different regression model (such as poisson regression with generalized estimating equations) would be more appropriate.*

**Answer:** Dear Editor, thank you for your comments and recommendations.

We have thoroughly reviewed the manuscript and provided further important details to clarify data sources, the main rationale and procedures for the statistical analysis, including the methods used in our sensitivity analysis. Indeed, the three study sites are geographically distant (400 – 552 Km between sites), in three different regions of the country with different administrative systems and different populations and therefore, the analyses were site-specific. The use of GEE as an alternative, would have been plausible if the analysis included the three cities at the same time. However, although the analysis is presented in single tables and figures to favour comparison across sites and to decrease the number of tables and figures, the analyses were site-specific. Please find below the details of the changes we made to clarify these points in the manuscript.

**Reviewer #1:**

Q.1.*The paper explores an important and well-known problem with dengue, chikungunya and Zika in Latin America. I liked the choice of comparing 3 different cities with varying levels of endemicity and thought that the concept of exploring under-reporting by sex, age, year and healthcare provider was interesting. However, I felt this concept wasn’t fully utilised. If under-reporting in respect of sex, age, year and healthcare provider had been explored for each pathogen then targeted suggestions could have been created. I feel it is really important to separate these factors by pathogen due to their differing host demographics. The paper would benefit from some clear hypotheses to investigate. This would then structure the result and discussion sections better for easier reading. The main concern for the paper is that the methodology is not clearly explained and I am not sure of the choice of statistical testing. This paper is well worth revising for resubmission as it would be interesting to see the concepts of the paper fully explored*.

**Answer No.1:** Dear Reviewer, thank you for your comments. We have clarified our research question in the introduction, we improved the explanation of the analysis plan, the rationale in the methods section, and finally provided further details about the determinants of underreporting in the discussion section. Although we believe that presenting the analyses by diseases is important and would provide insights about disease-specific determinants of reporting, the data used in this study (healthcare facility-diagnosed cases and surveillance-reported cases) presents several challenges for such approach. For instance, we acknowledge that confirmatory diagnosis of arboviruses is challenging in endemic settings and the risk for misclassification of the conditions -initially clinically diagnosed- is not negligible; which is the rationale for our sensitivity analysis. Another reason to present the overall assessment of the reporting comparing across diseases/conditions and not by disease was the need to harmonize the diagnosis system used at the healthcare facilities and those used in the surveillance system. This aspect as is explained in the methods (Lines 173-199; Lines233-255). In addition, to partially overcome the limitation presented by not presenting the analyses by disease, we still discussed some of the findings in light of the potential implications or explanations by each disease (Lines:388-410; Lines 416-429).

***Q.2****.Hypotheses are required to structure the paper. This will particularly help with the results and discussion sections. Specifically, it would be interesting to ask the question why the datasets are different. It is not clear from the paper whether the dominant hypothesis is that under-reporting is due to poor reporting to the national surveillance system, clinical diagnosis is poor or whether cross-reactivity/multiple infections are responsible*.

**Answer No.2:** Overall, underreporting in surveillance systems could be multifactorial. Factors related to the clinical presentation of the diseases and aspects related to the healthcare system could also affect the notification of diseases to surveillance systems. In the case of arboviral diseases in endemic settings, (under)reporting could be further affected by misclassification of the clinical diagnosis, given the similarities across conditions and challenges associated to laboratory confirmation. To clarify our hypothesis, we added a sentence at the end of the introduction:

Page 6, Lines: 140-147: “*We consider that individual characteristics and aspects related to health care access shape the reporting of this conditions in the Colombian context. To contribute to a better understanding of the reporting of dengue, chikungunya and zika in Colombia, we conducted a capture-recapture study of the arboviral diseases in three cities of Colombia from 2014 to 2017. In addition, given the possibility of misclassification of the diagnosis of arboviruses in endemic settings, we conducted a sensitivity analysis to explore the extent of misclassification in the notification of arboviruses in the study settings*.”

***Q.3.*** *In the methods section, I would like to see the expectations clarified. Do we expect the datasets to be identical if reporting has worked properly? Or is there something more complicated with the laboratory testing process? If a patient goes to a different healthcare facility, will it be reported in the 'recapture' but not the capture*?

**Answer No.3:** Ideally, under hypothetical perfect settings where clinical diagnostics is specific and sensitive enough, the capture and recapture datasets would be expected to be almost identical. However, in endemic settings with newly introduced pathogens and where laboratory diagnosis and confirmation are challenging, it is expected to have discrepancies to a certain degree. In endemic settings as Colombia, discrepancies are expected given limitations in laboratory confirmation and therefore the reluctance to report suspected cases.

Generally, if an individual seek care at a facility that is not part of the (study) capture settings, s/he would be reported in the recapture source but not in the capture setting. However, in our study, this was considered at the study design stage and therefore we used datasets from healthcare-networks/providers, rather than single institutions datasets when available or possible. We provided further details and paraphrased some sentences in the methods section as follows:

Page 8, Lines 187-189: “*We obtained four electronic datasets (two from Cali and one each from Medellin and Villavicencio), with registries of cases seeking care at the different collaborating healthcare facilities and surveillance data (SIVIGILA) from the Secretary of Health of each municipality*”

Page 8, Lines 196-225: “*Our capture sample was composed of all registries from patients who sought care at the collaborating healthcare facilities between January 1, 2014 to December 31, 2017 and with a healthcare facility’s diagnosis, according to the ICD-10 codes of undifferentiated fever, dengue, chikungunya, Zika, or non-specified vector-borne disease. In Colombia, individuals receive healthcare according to a health system coverage which is mainly divided into a subsidized system (for individuals without income or income below the minimum wage for which the government subsidized the healthcare attention) and the contributory system (for employees or individuals who could pay for the healthcare insurance)[29, 32, 44]. There are different healthcare providers or insurer in the country, each with healthcare facilities for different levels of attention within their network, providing care to users of either insurance system[44]. Given the possibility that individuals attend different healthcare facilities and in order to include a comprehensive capture sample, we used centralized data (main consultations and referrals) from the main healthcare providers in each city when available or possible. In Cali, we obtained data from a public/government owned network that provides care mainly to subsidized users in four healthcare facilities and the second dataset included data from all the healthcare facilities providing care to users from one of the main contributory insurance companies in the city. In Medellin, we used data from the main insurer in the city which provides healthcare attention to subsidized and contributory users in 10 main healthcare facilities, including data from a facility that mainly provide healthcare to users from the contributory system. In Villavicencio, we included the data from the main healthcare facility which is as well a university hospital and provide care to users from both subsidized and contributory insurance schem*e”

***Q.4.****The choice for statistical testing is unusual, potentially being an inappropriate choice. It is not fully explained, making evaluation difficult. The authors use a Poisson regression on a binary outcome predictor (recaptured or not)? More appropriate to consider a logistic regression with a binomial link function or a Poisson regression on the number of reported cases, with the number available as an offset*.

**Answer No.4:** Although the outcome (reporting =0 vs reporting =1) is binary and it is customary to use logistic regression, we tried to avoid some methodological issues associated to the logistic regression, including the overestimation of the odds ratio (OR) when the outcome is common due to the non-collapsibility of the OR [1, 2]. An alternative is the use of GLM using binomial regressions with a log-link, however, there are known issues with the lack of convergence, which we also experienced in this analysis [1-3]. We fit robust Poisson regression as an alternative to overcome the mentioned methodological issues related to the use of logistic regression, after considering the use of binomial regressions with a complementary log link, which converged but for which the interpretation is more complex and the implementation of the misclassification correction has not been assessed. Although the results from the logistic regression, the binomial regression with the complementary log (c-log) and the Poisson regression are consistent, we choose to present the results from the Poisson regression because those provide an unbiased effect estimate of the rate of reporting of arboviruses, without overestimating the rates. However, we acknowledge the possibility of some imprecisions in the estimation of the variance that do not affect the interpretation of the uncertainty of our rates -as confirmed by the analysis using other type of regressions. Since the analysis were site-specific, we did not use generalized estimating equations (GEE), which would have been the plausible alternative in case of conducting the overall underreporting analysis including the data from the three cities simultaneously[4]. We clarified this in the methods section (Page 10, line 265-270).

References:

1.	Chu H, Cole SR. Estimation of Risk Ratios in Cohort Studies With Common Outcomes: A Bayesian Approach. Epidemiology. 2010;21(6):855-62. doi: 10.1097/EDE.0b013e3181f2012b. PubMed PMID: 00001648-201011000-00017.

2.	Shrier I, Pang M. Confounding, effect modification, and the odds ratio: common misinterpretations. Journal of clinical epidemiology. 2015;68(4):470-4. doi: 10.1016/j.jclinepi.2014.12.012. PubMed PMID: 25662008.

3.	Zou G. A Modified Poisson Regression Approach to Prospective Studies with Binary Data. American Journal of Epidemiology. 2004;159(7):702-6. doi: 10.1093/aje/kwh090.

4.	Wang M. Generalized estimating equations in longitudinal data analysis: a review and recent developments. Advances in Statistics. 2014;2014.

Page 10, line 265-270: “*To obtain adjusted rates of reporting, we fit site-specific Poisson regressions with robust variance estimation to determine the predictors of the reporting and to estimate the adjusted probability of reporting by clinical diagnosis and year in each study site. We choose robust Poisson models over logistic regression to avoid overestimation of the rates given that the outcome is not rare, the non-collapsibility associated to the estimation of odds ratios, and lack of convergence of the binomial regressions with a log-link [46-48].”*

***Q.5****. I also think the presentation of the MC-SIMEX analyses is not ideal, it is presented as a magic black box. What does it do? How does it work? What are the assumptions? Please could you also clearly state the parameters used for the model*.

**Answer No.5.** The MC-SIMEX method is a tool developed to correct or assess the extent to which misclassification bias occurred. Given the opportunity of misclassification by clinical diagnostic (e.g., chikungunya or zika cases diagnosed as dengue) and the possibility of the misclassification affecting the reporting, we consider opportune to assess the potential effect of the misclassification in the reporting using the MC-SIMEX technique. We provided further details in the methods section as follows:

Page 10; lines 277- 294: ”…*we conducted a sensitivity analysis using simulation extrapolation for misclassification MC-SIMEX [48, 49]. In brief, the MC-SIMEX method is a tool developed to correct or assess the extent of the misclassification bias. The assessment and correction of the bias includes the extrapolation of the observed regression results from the adjusted model via simulation and correcting the reporting estimates using a correlation matrix with parameters of sensitivity and specificity for each possible diagnostic. In the simulation step, a degree or range of measurement error is applied to the naïve estimates (observed data), according to the provided information of specific/sensitivity of the observations. In the extrapolation step, the results are extrapolated back to an expected point of absence of measurement error [48-50]. The MC-SIMEX procedure for this study included the use of three sets of correlation matrix for each city: 1) a low sensitivity/specificity matrix were we set the correlation matrix between main diagnosis to 70%; 2) a high sensitivity/specificity matrix were we set the correlation matrix between main diagnosis to 90%; and 3) a correlation matrix using the observed data, this is, the actual correlation observed between the notified diagnosis and the institutional diagnosis, having the clinical diagnosis as the reference (Appendix 3 and 4). Parameter values of sensitivity/specificity were obtained from published reports in the literature and estimates from confirmed cases of analyzed data [12, 38]*.”

*Q.6.The wording ‘capture-recapture study’ invokes a specific type of analysis, so was surprised to see only a Poisson regression in the methods section*.

**Answer No.6.** In addition to what we had stated in the design and sources section (Wlines 173-175), we added a sentence in the data analysis section to refer to the specific analysis of the capture-recapture using Vong S et al., methodology and the respective equation as follows:

Page 10, line 257-263: “*To estimate the level of reporting we followed the approach described by Vong S, et al., where we estimate N, the estimated total number of arboviruses cases as follows:*

$$N=\left[ \frac{\left( N_{A}+1 \right)\left( N_{B}+1 \right)}{X_{AB}} \right]-1$$

*Where* $N_{A}$ *is the number of cases by the capture (from collaborating institutions),* $N_{B}$ *is the number of cases by the recapture (cases notified in SIVIGILA), and* $X_{AB}$*, is the number of cases reported by both, the capture and recapture. The multiplication factor (MF) is the inverse of underreporting rate and it is estimated by:* $MF=N/{N_{B}}$ “

**Reviewer #2:**

*Q.1. Can you provide any additional details on the healthcare facilities included in the study? Are they public/private? Do they serve particular populations? Do these facilities typically take patients with these diseases or would they have referred the patients to other facilities for confirmation or treatment? If they might refer patients, the facility might expect that other facilities would make the disease report*.

**Answer No.1:** Thank you for your comments. The health care facilities included in the study served both people under the subsidized insurance and people under the contributory insurance, we used mostly centralized data, so we obtained information by different level of attention, main consultations and referrals. We have provided the suggested details about the healthcare facilities in the methods section. Please refer to Pages 8-9, Lines 196-225 for the changes in the manuscript and to the Answer No.3 provided to Reviewer #1 for the excerpt of the manuscript that was modified in this document.

Pages 8-9, Lines 196-225: *“In Colombia, individuals receive healthcare according to a health system coverage which is mainly divided into a subsidized system (for individuals without income or income below the minimum wage for which the government subsidized the healthcare attention) and the contributory system (for employees or individuals who could pay for the healthcare insurance)[29, 32, 44]. There are different healthcare providers or insurer in the country, each with healthcare facilities for different levels of attention within their network, providing care to users of either insurance system [44]. Given the possibility that individuals attend different healthcare facilities and in order to include a comprehensive capture sample, we used centralized data (main consultations and referrals) from the main healthcare providers in each city when available or possible. In Cali, we obtained data from a public/government owned network that provides care mainly to subsidized users in four healthcare facilities and the second dataset included data from all the healthcare facilities providing care to users from one of the main contributory insurance companies in the city. In Medellin, we used data from the main insurer in the city which provides healthcare attention to subsidized and contributory users in 10 main healthcare facilities, we also included data from a facility that mainly provide healthcare to users from the contributory system. In Villavicencio, we included the data from the main healthcare facility which is as well a university hospital and provide care to users from both subsidized and contributory insurance scheme.*”

***Q.2****. Were individual models fit for each site or all together in one model? It seems they are all together but this could be more clear in the methods section*.

**Answer No.2**: Although the analysis is presented in single tables and figures to favour comparison across sites and to decrease the number of tables and figures, the analyses were site-specific. We have clarified this in the methods section.

Page 10, line 265-267: “*To obtain adjusted rates of reporting, we fit site-specific robust Poisson regressions to determine the predictors of the reporting and to estimate the adjusted probability of reporting by clinical diagnosis and year in each study site.*”

*Q.3. Why did you choose to evaluate age as a category instead of as a continuous variable? Why did you decide on these categories*?

**Answer No.3**: We obtained individual continuous age when possible. However, as mentioned in the footnote in Table 1, there was no individual information of age from the health facility data from Villavicencio. Therefore, to ensure comparability with other sites, we did dichotomize the variable age according to adults (>=18 years) or non-adults (<17 years). We added a sentence in the limitations section:

Page 18, line 502-503: *“…and the characterization of reporting by age was not possible due to incomplete individual age data from one healthcare facility …”*

*Q.4. lines 255-257: perhaps this sentence belongs in the methods section.*

*Figures are very nice*.

**Answer No.4**: Thank you for the comment. We removed the sentence from the results section and rephrased the method section.

Page 11, lines 286-288: “*To test the robustness of the stimulation estimates, the MC-SIMEX procedure in this study included the same sets of three correlation matrixes for each city*…”

*Q.5. For instances when the healthcare diagnosis differed from the SIVIGILA diagnosis, how could this occur? Does the reporting agency make a final reporting diagnosis that might differ from the ICD-10 final clinical diagnosis? Does SIVIGILA review case details for non-specified vector-borne diseases? Does SIVIGILA coordinate with some national testing service data or other test results*?

**Answer No.5**: Instances for which discrepancies in diagnosis may occur are related to several aspects. First, the presence of unconclusive laboratory results, e.g., a positive serologic test for both dengue and zika, without a confirmation test such PCR or PRNT could be understood as an “unspecified arboviruses”. The “unspecified arbovirus” diagnostic has an ICD-10 code but could not be registered in SIVIGILA as such. SIVIGILA only allows the use either dengue, chikungunya or Zika as diagnosis. Other aspects include the use of clinical presumptive diagnostics before confirmation in the clinical practice. According to the protocols for surveillance of arboviruses in Colombia, the cases reported as either dengue, chikungunya or Zika should be notified using the pre-specified codes for each condition. While the ICD-10 could provide different entries attributable to dengue (A90, A90x; A94x), SIVIGILA’s code allow the entry of any case diagnosed as dengue irrespective of the ICD code.

SIVIGILA does not review the non-specified cases, because it does not include it as a diagnostic in the surveillance system. However, the national surveillance system allows the revision of the notification by allowing the institutions to make adjustments when the cases are discarded, or when another cause of disease is confirmed. The national surveillance system includes in the protocol the serotyping and confirmation of a subset of the reported cases by testing those sample at provincial and national laboratories. Clarifications about the process are included in the design and sources section (lines 175-182 and lines 227-229) and in the data analysis section (lines 249-251).

*Q.6. Reporting error? Can you discuss how these differences might arise*?

**Answer No.6**: In addition to the aspects mentioned in the previous answer, reporting error could occur from centers where lab resources are scarce and no confirmation is made or where the reporting is not made due to lack of trained personnel or any staff to handle the notification process where it is not automatized. Likewise, during outbreaks, overcrowded healthcare facilities may not prioritize the reporting, or this would be incomplete, therefore a proportion of cases will not be reported to the surveillance system. To avoid issues inherent with the dataset, we used data from SIVIGILA that reflected the adjustments (i.e., data where cases discarded, duplicated, or inconsistent entries were removed).

*Q.7. Iines 262, 312-316: “…type of healthcare provider” / “difference observed between public and private healthcare providers” I think it is difficult to pinpoint exactly what this effect is, since you have a single healthcare facility for each city (except for Cali) and that isn’t enough healthcare facilities to determine the impact of type of healthcare facility. For your dataset, you can’t really distinguish the impact of city/healthcare facility, as these are essentially the same thing in your data (i.e. one facility per city). It could just be that the particular facility is better/worse at reporting with it not being related to being public or private*.

**Answer No.7**: We have provided more details about the healthcare providers structure and the data sources in Colombia in the methods section to clarify this point. In Cali, the data form the subsidized system (public) were from 4 institutions and the data from the contributory insurance (private) is as well from a network with different facilities. Thus, the interpretation was made as for the providers rather than to the facility. We have revised the manuscript and consistently used the term “subsidized” and “contributory” for public and private institutions respectively.

Pages 8 and 9, lines: 199-225: “*In Colombia, individuals receive healthcare according to a health system coverage which is mainly divided into a subsidized system (for individuals without income or income below the minimum wage for which the government subsidized the healthcare attention) and the contributory system (for employees or individuals who could pay for the healthcare insurance) [29, 32, 44]. There are different healthcare providers or insurer in the country, each with healthcare facilities for different levels of attention within their network, providing care to users of either insurance system. Given the possibility that individuals attend different healthcare facilities and in order to include a comprehensive capture sample, we used centralized data (main consultations and referrals) from the main healthcare providers in each city when available or possible. In Cali, we obtained data from a public/government owned network that provides care mainly to subsidized users in four healthcare facilities and the second dataset included data from all the healthcare facilities providing care to users from one of the main contributory insurance companies in the city. In Medellin, we used data from the main insurer in the city which provides healthcare attention to subsidized and contributory users in 10 main healthcare facilities, including data from a facility that mainly provide healthcare to users from the contributory system. In Villavicencio, we included the data from the main healthcare facility which is as well a university hospital and provide care to users from both subsidized and contributory insurance scheme*.”

Line 267: “*The adjusted model included individual level covariates including sex, age, type of diagnostic test, and health care provider (subsidized or contributory) information, when available*.”

Line 432: “*The differences observed between mainly subsidized or mainly contributory healthcare providers in Cali were mostly of magnitude of effect*.”

*Q..8. line 25 and throughout: Zika is always capitalized line 182: change “by” to “at*”

**Answer No.8**: We reviewed the manuscript and did capitalized Zika throughout the document and the preposition was changed accordingly.

Line 241: *“…given that at the moment of its introduction, Zika did not have assigned an ICD-10 code [33, 45]*.”

*Q..9 Can you clarify in the intro that non-specified vector-borne disease is not reported to SIVIGILA*

**Answer No.9**: We had a sentence in the introduction indicating that report of dengue, chikungunya and Zika is mandatory. Line 138-139: “*the mandatory reporting of dengue, chikungunya, and Zika to the National Surveillance System (SIVIGILA) in Colombia [32-34]*,”. However, we stress this point in the methods section as well:

Line 249: “*SIVIGILA does not allow the report of “unspecified arboviruses” or “non-specified vector-borne diseases”, therefore the institutions used the dengue, chikungunya, or Zika respective code from SIVIGILA to report the cases [32-34, 45].”*

**Reviewer #3:**

*Q.1. The objectives of the study are clear and the study design is appropriate (using method previously published); the sample size is adequate as well.*

*It would be helpful for the methods section to better explain the statistics specifically related to capturerecapture and define the outputs in the Results section more carefully*.

**Answer No.1**: Thank you for your comments. We have provided more details about the methods and structured better the results and discussion section. Detailed answers are provided below.

*Q.2.Even though it is following Vong et al., it would be nice to have some of the basics briefly revisited in the methods section--it's only mentioned in the first sentence of the Methods. For example, are you using Vong's method to estimate the "true" total number of dengue/chik/Zika cases? I don't see that in the Results section at all, but that would be very interesting. While this can be implied from the reporting percents that you show, it could be nice to show the estimated numbers as well. Or maybe I missed that in the results section*?

**Answer No.2**: Thank you for pointing this out. We have added a paragraph with further details about the method proposed by Vong et al., in the methods section (Page 10, line 257-263; described in answer No.6 to reviewer #1). Since the estimation of the “true” number of cases is unadjusted and include the diagnostics reported from the healthcare facilities, which included some limitations in the confirmation, the estimation of the multiplication factors are included in the results section but the estimation of the “true” number of cases is included in the appendixes.

Likewise, we have included a sentence in the results section, discussion and appendix (Table S3A and Table-S3B) as follows:

Line 334 – 338: “*The overall -unadjusted- multiplication factors (MF) obtained from the capture-recapture analysis following Vong’s approach were 8.7, 6.8 and 18.9 for Cali, Medellin and Villavicencio, respectively. Across arboviruses, the smallest unadjusted MFs were for dengue: MF= 2.5 in Cali; MF=5.9 in Medellin, and MF= 2.03 in Villavicencio; while the highest were for chikungunya in Cali (MF=38.2) and Medellin (MF=8.6) and for Zika in Villavicencio (MF=110.6) (Appendix 3)*.”

Lines 431-437: “*Results from the crude proportion of reporting, the multiplication factors for notification from the capture-recapture analysis and the estimated adjusted rates of reporting are consistent. These finding indicate higher proportion of reporting in Medellin, followed by Cali and Villavicencio and therefore smaller multiplication factors for reporting in Medellin and Cali, compared to Villavicencio. Likewise, dengue reporting seems higher in all sites, which is consistent to smaller multiplication factors for dengue reporting compared to chikungunya and ‘Zika/non-specified vector-borne diseases’*.”

**Appendix 3.A:**

**Table S.3A.** Estimation of overall -unadjusted- total number of cases, and multiplication factors for notification by city.

| **City** | **Capture (N_A_)** | **Recapture (N_B_)** | **Reported (X_AB_)** | **Estimated Total cases (N)** | **Multiplication Factor**  **(MF)** |
| --- | --- | --- | --- | --- | --- |
| **Cali** | 97,193 | 75,379 | 11,223 | 652,808.7 | 8.7 |
| **Medellin** | 32,437 | 28,689 | 4,760 | 195,512.9 | 6.8 |
| **Villavicencio** | 18,121 | 14,734 | 961 | 277,863.4 | 18.9 |

*N* is the estimated total number of arboviruses cases $N=\left[ \frac{\left( N_{A}+1 \right)\left( N_{B}+1 \right)}{X_{AB}} \right]-1$

$N_{A}$ is the number of cases by the capture (from collaborating institutions), $N_{B}$ is the number of cases by the recapture (cases notified in SIVIGILA), and $X_{AB}$, is the number of cases reported by both, the capture and recapture. The multiplication factor (MF) is the inverse of underreporting rate and it is estimated by: $MF=N/{N_{B}}$

**Appendix 3A: Table S.3B.** Unadjusted multiplication factors for notification of arboviral conditions by site.

| **Diagnostic** | **Multiplication Factors (MF)** | | |
| --- | --- | --- | --- |
| **Site** | **Cali** | **Medellin** | **Villavicencio** |
| Dengue | 2.5 | 5.9 | 2.0 |
| Chikungunya | 38.2 | 8.6 | 4.3 |
| Zika /other vector-borne diseases | 2.7 | 0.7 | 110.6 |

*Q.3. How is rate of notification and IRR that are referred to in Results related to this capture-recapture method? It's not clear how all the statistics that are done fit together into one picture. Also, the sensitivity analysis referred to in the Results section is not well explained in Methods. So, to summarize, the statistical methods need to be significantly expanded and more detail given*.

**Answer No.3**: Overall, we estimated crude proportion of overlapping reporting, estimated the unadjusted level of reporting and multiplication factors (MF) for reporting following Vong’s approach and estimated the adjusted rates of reporting using the Poisson regressions with robust variance estimation. The rate of reporting and the respective rate ratios (RR) and the capture-recapture relate because the rate of reporting is derived from the identification of cases reported in both sources. The sensitivity analysis with the MC-SIMEX was intended to assess the extent of the misclassification bias in the clinical diagnosis for arboviral reporting in each site. We clarified and provided additional details in the methods and results section.

Please refer to lines: 257- 271 in the methods section, as it has been described in answers 4, 5 and 6 to Reviewer # 1.

Line 340: We consistently use the term rate ratio (RR) throughout the text.

*Q.4. The analysis results don't match what is described in the Methods section, so it is hard to see how everything fits together. The data and conclusions about under-reporting, misclassification, and variation by year and location are very interesting*.

**Answer No.4**: We reviewed the organization and presentation of the methods to clarify this point. Likewise, we have improved the flow of the text and consistency of information by providing the details in methods (lines: 257- 292), Results (Lines 333 – 338), and discussion (Lines 428-433) as it has been described in answers 4, 5 and 6 to Reviewer # 1 and in answer No.2 for Reviewer #3

*Q.5.Minor comments:*

*For Table 2 are the Dengue Chikungunya Zika columns total across all years*?

**Answer No.5**: Yes, those are the number of reported cases in both sources during the study period as mentioned in the table heading.

*Q.6. Lines 250-257 are not clear--I can't find an explanation of the sensitivity analysis in the Methods. Are you referring to MC-SIMEX*?

**Answer No.6**: Yes. We have added further details to the explanation of the sensitivity analysis in the methods section and clarify the sentence in the results sections as follows:

Lines 275-295: “*Given the possibility of misclassification of clinical diagnosis of arboviruses (e.g., diagnosing chikungunya or Zika cases as dengue at early stages of its introduction) [1, 11, 13, 15, 29, 37], we conducted a sensitivity analysis using simulation extrapolation for misclassification MC-SIMEX [49, 50]. In brief, the MC-SIMEX method is a tool developed to correct or assess the extent of the misclassification bias. The assessment and correction of the bias includes the extrapolation of the observed regression results from the adjusted model via simulation and correcting the reporting estimates using a correlation matrix with parameters of sensitivity and specificity for each possible diagnostic. In the simulation step, a degree or range of measurement error is applied to the naïve estimates (observed data), according to the provided information of specific/sensitivity of the observations. In the extrapolation step, the results are extrapolated back to an expected point of absence of measurement error [49-51]. To test the robustness of the stimulation estimates, the MC-SIMEX procedure in this study included the same sets of three correlation matrixes for each city: 1) a low sensitivity/specificity matrix, were we set the correlation matrix between main diagnosis to 70%; 2) a high sensitivity/specificity matrix, were we set the correlation matrix between main diagnosis to 90%; and 3) a correlation matrix using the observed data, this is, the actual correlation observed between the notified diagnosis and the institutional diagnosis, having the clinical diagnosis as the reference (Appendix 3 and 4). Parameter values of sensitivity/specificity were informed by published reports in the literature and estimates from confirmed cases of analyzed data [12, 38].”*

Lines 372-373: “*The sensitivity analysis results from the MC-SIMEX showed that the reporting estimates were likely affected by the misclassification of each arboviral disease*.”

*Q.7. The conclusions are supported by the data and analysis and limitations are clear. I think there could be more discussion about how to use this to support forecasting and mathematical modeling by providing reporting rates and thinking about if there are any generalizations that can be made into the future on reporting rates*.

**Answer No.7**: We agree that it is important to use these findings to inform forecasting and mathematical modeling exercises. Indeed, this study was conducted as a preliminary analysis to assess the scope of underreporting to inform future studies on disease forecasting in the country. We added a short sentence in the discussion section as follows:

Line 470-481: “*These findings pose an important challenge for estimating the arboviral disease burden in Colombia[4]. Importantly, these findings are useful as well to inform forecasting and mathematical modeling exercises in the country and other Latin American endemic settings with similar passive surveillance systems, where the notification of cases does not rely entirely on laboratory confirmation.”*

*Q..8. It would also be helpful to better explain the implications of the MC-SIMEX analysis. For example, lines 318-320 are not clear to me--are you saying that you are under-estimating the reporting rates so that reporting is actually better/higher than what you are estimating*?

**Answer No.8**: We have clarified the methods in the text and as indicated in the figure legends, the results indicate that the observed results from the adjusted regression model, tend to under-estimate the rate of reporting, due to the potential misclassification in the diagnosis. The higher estimates from the MC-SIMEX correspond then to the “correction” and extrapolation made by the simulation procedure. We expect that the changes in the methods (Lines 275-295), results (lines 372-377) and discussion (lines 460-464).

Lines 460-464: “*As anticipated, our simulation extrapolation assessment of misclassification revealed that there was a potential underestimation of arboviruses case reporting. The simulation portion showed that increasing misclassification error moved the estimated reporting rates further towards the null. Consequently, the “error-free” extrapolated estimates were on average, larger than the adjusted but potentially misclassified estimates*.”

*Q.9. Lines 322-323 are also not clear*.

**Answer No.9**: Thank you for pointing this out. There was indeed a typo/missing word. We have made the respective changes.

Line 466-468: “*Across diseases, the potential underestimation of reporting was observed more in chikungunya and for Zika/‘non-specified vector-borne diseases’ diagnostics than for dengue*.”

*Q.10. In line 281 are you referring to the reporting rate as determined by your capture-recapture stats? It seems like the word reporting is perhaps used differently in different parts of the manuscript*...

**Answer No.10**: We were referring to the adjusted rate of reporting obtained by the regression model. Line 428- 432 makes reference to the integration of all results from the crude proportions, the capture-recapture results and the results from the regression. We have revised the manuscript to ensure consistency and clarified the sentence as follows:

Line 409: “*Our study showed a differential adjusted rate of reporting by year, which was expected given the endemo-epidemic nature of the arboviruses [1, 2, 29, 38]*”

*Q.11. Zika should be capitalized line 218 what do you mean by "proportion of reporting" is that proportion of all reported cases*?

**Answer No.11**: Zika: We reviewed the document and made the changes accordingly. “Proportion of reporting”: yes, it the proportion of reported cases from those diagnosed at healthcare facilities.

*Q.12. line 221 what do you mean by "proportion of diagnosis agreement"*?

**Answer No.12**: We meant the agreement between data sources about a given diagnosis. For example, from all healthcare facility-diagnosed dengue cases, how many of those were reported in SIVIGILA as dengue, as well. We have revised the phrase as follows:

Line 327: “*diagnosis’ agreement*”

*Q.13. line 229 what do you mean by "notification"? is that different from reporting*?

**Answer No.13**: The words notification and report were used interchangeably. However, we reviewed the manuscript and used consistently the term “reporting” throughout.

*Q.14. please define IRR line 236 what do you mean by "adjusted predicted probability of reporting"? adjusted by age only*?

**Answer No. 14**: We have reviewed the document and consistently used the term rate ratio (RR) throughout the text. The adjusted probability was estimated form the regression model. We clarified the sentence about the estimated probability and as follows:

Line 352-353: “*The estimated adjusted probability of reporting any given clinical diagnosis, obtained from the regression model, changed by year in each study site (Figure 2).*”

*Q.15. The manuscript could generally benefit by clarification throughout. The data and analysis is very interesting and could be useful both in public health and modeling applications*.

**Answer no.15**: Thank you for your comments. We have revised the manuscript and incorporated the respective changes to improve clarity and ease of reading.
